# Patient-reported quality of outpatient healthcare in patients with chronic back or arthrosis pain with long-term opioid therapy in Germany

**DOI:** 10.1186/s12875-025-02881-3

**Published:** 2025-06-21

**Authors:** Anja Niemann, Nils Frederik Schrader, Christian Speckemeier, Carina Abels, Nikola Blase, Milena Weitzel, Anja Neumann, Cordula Riederer, Joachim Nadstawek, Wolfgang Straßmeir, Jürgen Wasem, Silke Neusser

**Affiliations:** 1 Institute for Healthcare Management and Research, University Duisburg-Essen, Thea-Leymann-Str. 9, 45127 Essen, Germany; 2 DAK-Gesundheit, Nagelsweg 27, 20097 Hamburg, Germany; 3 Association of German Doctors and Psychotherapists Practicing in Pain Medicine and Palliative Care (BVSD e.V.), Katharinenstraße 8, 10711 Berlin, Germany

**Keywords:** Healthcare quality, Outpatient healthcare, Chronic pain, Opioid analgesics, Back pain, Arthrosis pain, Questionnaire, PACIC-5A, Germany, Guideline adherence

## Abstract

**Background:**

Managing patients with chronic non-cancer pain (CNCP) in outpatient healthcare is challenging. Long-term opioid therapy is an option for treatment responders with guideline recommended indications. However, opioid use poses risks of severe side effects, including misuse, and therefore needs to be integrated into a high-quality healthcare process. This analysis evaluates the association between healthcare quality according to the evidence-based Chronic Care Model (CCM) in the treatment process of patients receiving long-term opioid therapy for chronic back and/or arthrosis pain, and patient-related or healthcare related variables.

**Methods:**

A cross-sectional patient survey was sent to a random sample of 3,037 individuals with long-term opioid therapy and chronic back and/or arthrosis pain insured by a large nationwide German statutory health insurance. Healthcare quality according to the CCM was assessed by the Patient Assessment of Chronic Illness Care (PACIC-5A) questionnaire. Internal reliability of the assessment instrument was determined using Cronbach’s α. Descriptive analysis of the outcome scales were conducted, alongside subgroup analyses considering patient characteristics, patient’s health situation, and pain treatment aspects. Testing for statistical significance was performed by Mann-Whitney U test and Kruskal-Wallis test. Effect sizes, namely Eta and Spearman’s Rank correlation coefficient, were calculated.

**Results:**

The analysis included 661 individuals. Participants were predominantly female (76%) with an average age of 69 years (SD 12.5). PACIC-5A score ratings across all (sub)scales were low, with a summary score rating of 2.4 (on a scale ranging from 1 (worst) to 5 (best)). Positive correlations with treatment quality were observed in the subgroup analysis concerning guideline-compliant pain treatment aspects such as setting therapy goals or a comprehensive treatment concept. Patient characteristics showed little to no correlations, except for a positive correlation between higher PACIC-5A rating and both lower age and higher education. Patient’s health situation presented a mixed picture, with no clear correlation between pain intensity/impairment, and PACIC-5A scores.

**Conclusions:**

The provision of healthcare for patients with long-term opioid therapy for CNCP seems to be inadequate according to the CCM. Guideline-recommended pain treatment aspects exhibited a positive correlation with healthcare quality according to CCM. Enhancing the implementation of the CCM in the outpatient healthcare process may improve healthcare quality.

**Trial registration:**

German Clinical Trials Register, DRKS00024854. Registered 04/28/2021.

**Supplementary Information:**

The online version contains supplementary material available at 10.1186/s12875-025-02881-3.

## Background

In Germany, chronic pain is a widespread condition [1] and is one of the main reasons for seeking outpatient medical care [2]. Back pain and arthrosis are among the most common pain disorders. According to a survey representative for the German adult population, the prevalence of chronic back pain was 15.5% in 2019/2020 [3]. Arthrosis, which is the most common joint disease worldwide and is characterized by pain as a leading symptom, is also a common cause of chronic pain [4] with a 12-month prevalence of 18% in the adult German population in 2014/2015. This prevalence sharply increases with age [5].

Opioids are one possible option for the treatment of severe chronic non-cancer pain [6]. However, opioids bear the risk of strong side effects, including misuse. At the time of the survey, opioids were not recommended as a first-line therapy in the German evidence-based, indication-specific guidelines for gonarthrosis and coxarthrosis. However, a short-term use of low potency opioid agents in inoperable patients or as a bridge until surgery if non-opioids are contraindicated or ineffective may be considered [7]. The guideline on gonarthrosis has since been updated. The recommendation has changed in particular to the effect that long-term therapy (> 3 months) can be recommended if patients report significant pain reduction and good tolerability in short-term therapy [8]. Likewise the German evidence-based indication-specific guideline on non-specific lower back pain does not recommend opioids as a primary therapy option [9]. An evidence-based guideline for the long-term opioid therapy for chronic non-cancer-related pain (LONTS – Langzeitanwendung von Opioiden bei chronischen nicht-tumorbedingten Schmerzen) is available for Germany, also recommending optimization of non-drug and non-opioid alternatives before initiating opioid therapy [6]. If opioid therapy is initiated after careful consideration of all treatment options, the LONTS guideline mentions both chronic back pain and arthrosis pain as possible medical indications for long-term opioid therapy [6]. The guideline recommends the integration of opioid therapy in chronic non-cancer pain into a broad therapeutic concept, which includes setting therapy goals and complementary interdisciplinary therapy, such as physical therapy, psychotherapy, or self-help groups [6].

As part of the Op-US project (Opioid Analgesics - trends in opioid care for chronic non-cancer pain in Germany, Innovation Fund of the Joint Federal Committee of Germany, grant number 01VSF19059), a patient survey was conducted among individuals with chronic back and/or arthrosis pain who received a long-term opioid therapy.

It is assumed that the examined patient group is exposed to severe stress and impairment in everyday life due to pain associated with their underlying disease that requires long-term opioid therapy. Consequently, the analysis of healthcare quality within the context of a chronic illness in outpatient healthcare is of interest. In Germany, opioids are prescribed as part of outpatient care, which is predominantly provided by general practitioners. A high-quality treatment in outpatient healthcare of a complex chronic illness is challenging which includes the traditionally still rather passive role of the patient, the focus on acute medicine, increasingly complex diagnostic as well as therapeutic options, and the efficient coordination of several service providers [10]. This issue is addressed by the concept of the chronic care model (CCM) providing evidence-based recommendations to improve the quality of healthcare. The objective is to facilitate productive patient-provider interactions that lead to better functional and clinical outcomes by improving the organization of the outpatient healthcare provider and involving the community. In addition, patients are empowered by healthcare providers to actively manage their condition themselves in an informed manner. Furthermore, the practice team is empowered by knowledge and sufficient resources to act rather than react, so that they can provide effective clinical healthcare and positively influence patient behavior [11, 12].

The aim of this analysis was to survey the quality of outpatient healthcare according to the CCM, which includes guideline-compliant pain treatment aspects [6], from the perspective of patients with chronic back and/or arthrosis pain and with long-term opioid therapy. Furthermore, exploratory subgroup analyses were applied.

## Methods

The analysis was based on a cross-sectional survey of a random sample of patients with long-term opioid therapy for the medical indications back and/or arthrosis pain. These patients were insured by “DAK-Gesundheit”, a statutory health insurance (SHI) company operating nationwide in Germany. Regarding the definition of long-term opioid therapy, different time periods are applied in the literature [13]. A period of ≥ 90 days is a common definition for long-term opioid therapy, also consistent with the German guideline [6, 13]. Therefore, it will be used in this evaluation. The sample was selected via administrative claims data. Inclusion criteria encompassed at least one opioid prescription in the first and second quarter of 2020 (the first and second prescription had to be at least 90 days apart), age over 17 years, and evidence of chronic back pain (International Classification of Diseases – 10th Revision – German Modification (ICD-10-GM) M42.16-19, M42.90, M42.96-99, M43.0-1, M47.26-27, M47.29, M47.86-88, M47.99, M48.06, M48.2, M54.16, M54.5, M55.3, M.99.33; M99.43, M99.53) and/or arthrosis pain (ICD-10-GM M15-M19) in 2020. ICD-10-GM codes were chosen based on the LONTS guideline specification [6] for possible indications for long-term opioid therapy. Exclusion criteria comprised evidence of cancer and palliative care (see study protocol for exact operationalization [14]). Due to the 9-month delay in outpatient administrative claims data, the cohort selection can only occur with a corresponding time lag.

In November 2021, a standardized questionnaire was sent to a random sample representative of those insured by “DAK-Gesundheit”, who were eligible according to the defined in- and exclusion criteria. Included were opioid agents with the Anatomical Therapeutic Chemical Classification System (ATC) codes N02AA01, N02AA03, N02AA05, N02AA55, N02AB03, N02AE01, N02AX02, N02AJ13, N02AX06. After sampling, individuals were excluded if they were under legal guardianship or had a postal address abroad. In April 2022 the standardized questionnaire was sent to a further sample. Opioid agents with the ATC code N02AX51 were included. The second sample was drawn since the ATC code N02AX51 was not included in the first sample and to increase the response rate.

Insured persons who returned a sufficiently completed questionnaire, defined as total number of missing values < 208 of possible 267 missing items, were included. It must be emphasized that only a small part of the overall questionnaire was used for the present analysis (see Additional file 1). A further inclusion criterion was the presence of a signed consent form. Individuals who did not indicate either back or arthrosis pain in the questionnaire (question 1 of the survey - see Additional file 1) were excluded. Moreover, questionnaires not allowing a distinct pseudonymization were excluded. All analyses refer to the total study sample, regardless of the active ingredient that led to their inclusion. Administrative claims data of “DAK-Gesundheit” from the year 2020 were linked to the survey data of the insured persons included. Further details of the methodology can be accessed in the published study protocol [14].

**Fig. 1:**
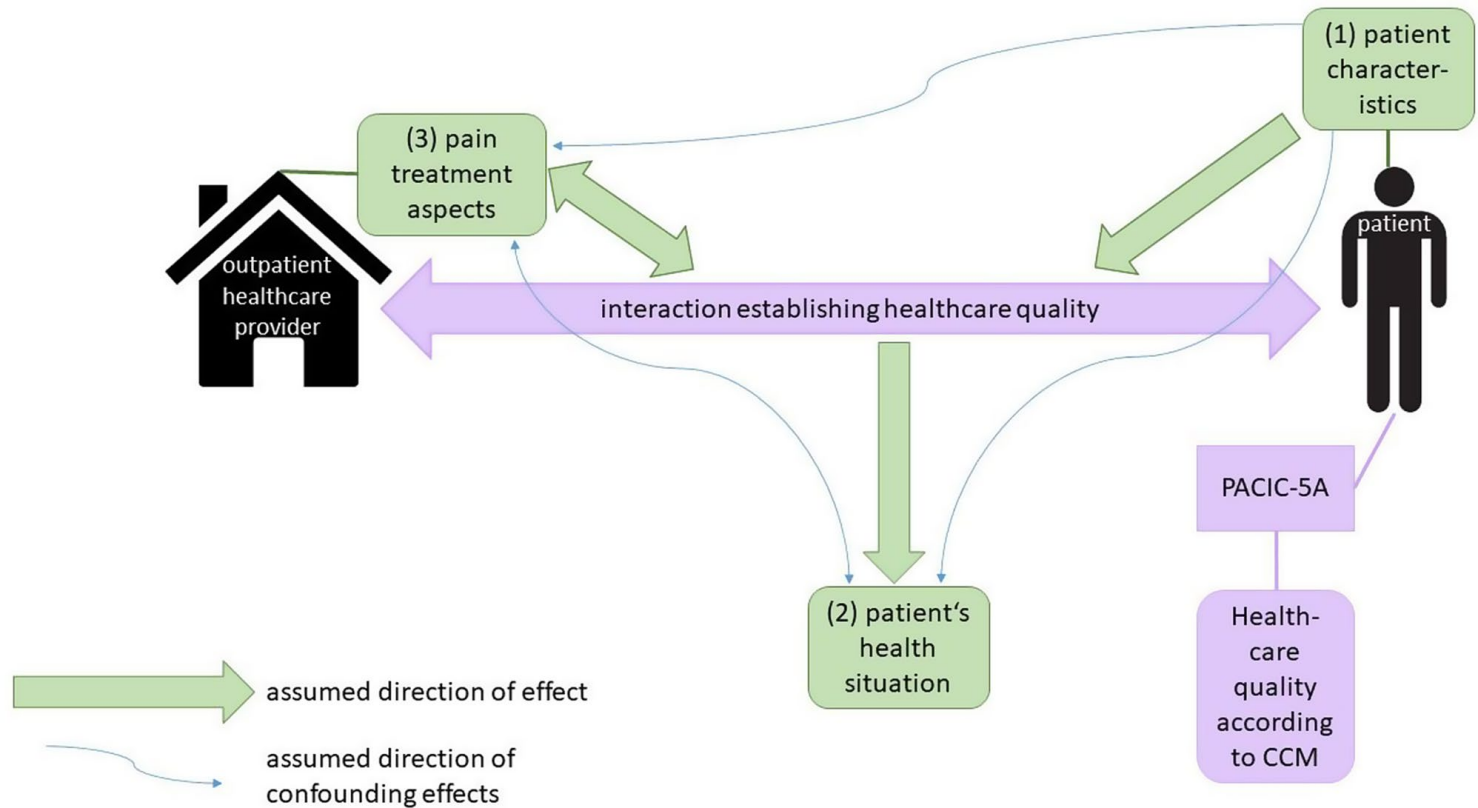
*Assumption on the direction of effect. Own illustration*,* based on Glasgow et al. (2001)* [12]. (1). patient characteristics: age, sex, migration background, pain diagnosis, highest educational qualification (2). patient’s health situation: psychological distress, symptoms of opioid Substance Use Disorder, severity of pain intensity and degree of disability (3). pain treatment aspects: outpatient pain therapy, setting of therapy goals, comprehensive treatment concept, categorized procedures of interdisciplinary pain therapy

For this analysis, we hypothesized three categories of effects in the context of quality of healthcare according to the CCM (see Fig. 1). First, we assumed that patient characteristics, such as age or education, act as independent variables possibly affecting the provided quality of healthcare since healthcare in accordance with the CCM is the product of an interaction and this interaction might be influenced by patient characteristics. It is also conceivable that patient characteristics influence the perception of healthcare quality according to the CCM. Second, patient’s health situation, e.g., psychological distress or pain intensity, were expected to be dependent variables improving with high quality of healthcare as specified by the CCM. Last, we took a closer look at selected parts of the healthcare process, such as certain therapies or agreements between physician and patient. This category is referred to below as “pain treatment aspects”. We expected that pain treatment aspects influence healthcare quality according to the CCM, with the direction of effect being bidirectional. For instance, in an overall high quality outpatient healthcare setting, there is a greater likelihood of establishing therapy goals. Moreover, when therapy goals were set, the chances of a good rating of healthcare quality in terms of the CCM are higher. We hypothesized that pain treatment aspects, which are in line with LONTS guideline [6] recommendations and supplementary consultation of specialized pain physicians are accompanied with a better quality in healthcare as per the CCM. Figure 1 displays the assumed direction of confounding effects that must be taken into account when interpreting the results.

The standardized questionnaire (an excerpt of the questions relevant to the present analysis can be found in Additional file 1) included questions utilized in the German pain questionnaire [15] and the German Epidemiological Addiction Survey 2015 [16], items derived from recommendations of the German evidence-based guideline LONTS [6], and additional validated survey instruments [17–19]. Additional file 2 provides a summary of information on the variables of the questionnaire and administrative claims data included in this analysis. This includes the assessment of healthcare quality according to the CCM [18], patient characteristics, patient’s health situation and pain treatment aspects (see also categorization in Fig. 1).

The quality of healthcare according to the CCM was evaluated using the “Patient Assessment of Chronic Illness Care” (PACIC) questionnaire, which was designed to operationalize the assessment of the quality of outpatient healthcare according to the CCM from the patient’s perspective [20]. A culturally and linguistically adapted, and validated version of the questionnaire is available for Germany [18]. The 5 A model (PACIC-5A) comprises an evidence-based and patient-centered approach to counseling regarding patient behavior change, based on the CCM [21]. Participants were asked to refer to the opioid therapy for medical treatment during the last six months. If their last visit was more than six months ago, they were asked to recall the last visit to the physician who administered their opioid therapy. Twenty-six questions were answerable using a 5-point Likert scale (1 “almost never” to 5 “almost always”). Using the scoring instructions provided by Rosemann, Laux [18], the “5A summary score”, which excludes question item 5, was calculated, resulting in the subscores “assess”, “advise”, “agree”, “assist” and “arrange”. The maximum allowable missing item count per subscore was one. Subscores were calculated as mean of the five underlying items. In case of one missing value, the mean of the remaining four items was imputated. To compute the total score, all corresponding subscores of an insured person needed to be available. Table 1 elucidates the meaning of the subscores of the 5 A-model [22].

**Table 1:** Five subscores of the 5A model of the patient assessment of chronic illness care evaluation

5A items	Explanation according to Whitlock(2002) [22]
assess	“Ask about/assess behavioral health risk(s) and factors affecting choice of behavior change goals/methods.”
advise	“Give clear, specific, and personalized behavior change advice, including information about personal health harms and benefits.”
agree	“Collaboratively select appropriate treatment goals and methods based on the patient’s interest in and willingness to change the behavior.”
assist	“Using behavior change techniques (self-help and/or counseling), aid the patient in achieving agreed-upon goals by acquiring the skills, confidence, and social/environmental supports for behavior change, supplemented with adjunctive medical treatments when appropriate […].”
arrange	“Schedule follow-up contacts (in person or by telephone) to provide ongoing assistance/support and to adjust the treatment plan as needed, including referral to more intensive or specialized treatment.”

The calculation of the severity of psychological distress via the Patient Health Questionnaire-4 (PHQ-4) [19] incorporated two Diagnostic and Statistical Manual of Mental Disorders, Fifth Edition (DSM-V) diagnostic core criteria for depressive disorders and two core criteria for generalized anxiety disorder [17]. Missing values were not allowed. The extent of opioid addiction was assessed via a modified section of the German Epidemiological Addiction Survey 2015 [16]. Results were classified into the variable expressions for the addiction problem, ranging from “none” to “severe”, by assigning the response items to nine DSM-V diagnostic criteria for Substance Use Disorders [23]. Whether a DSM-5 criterion applies is assessed if at least one question related to this criterion is not missing. For the calculation of the sum score, the classification is based on, the underlying nine criteria must not be missing. A detailed description of the scoring method can be found in Additional file 3. Pain intensity and pain-related impairment were assessed via the Graded Chronic Pain Scale (GCPS) [24] version of the anamnesis survey of the German pain questionnaire [15] and evaluated according to the method of the pain questionnaire manual [25]. Missing values were not allowed.

IBM SPSS Statistics 29.0 was utilized for data analysis. The results of the PACIC-5A were presented descriptively as mean values and standard deviations. Additionally, the internal consistency was assessed using Cronbach’s α, with a value exceeding 0.7 indicating acceptable correlation [26]. Furthermore, floor and ceiling effects were described. For the 5 A scores, subgroup mean values were calculated for the variables presented in detail in Additional file 2. Moreover, the statistical significance of subgroup differences was tested for ordinally or metrically scaled independent variables using the Spearman-Rho test, for dichotomously scaled independent variables using the Mann-Whitney U test, and for polytomous nominally scaled independent variables using the Kruskal-Wallis test. For all metrically scaled independent variables that were converted to ordinal/dichotomous scaling for the subgroup presentation of means, the underlying metric variable was used to calculate the significance levels of the group differences. The statistical significance level for all analysis was set at 0.05. Furthermore, effect sizes for nominally scaled variables were calculated using Eta, and for ordinally or metrically scaled variables using Spearman’s Rank correlation coefficient. To account for individual questions of the PACIC questionnaire, the frequencies of the response options and the mean values of the individual questions were described.

## Results

Of the 4.9 million insured adults of “DAK-Gesundheit” in 2020, 34,898 (0.71%) individuals in the first selection (without tilidine/naloxone) met the in- and exclusion criteria. In the second selection (only tilidine/naloxone), 28,232 (0.58%) insured met in- and exclusion criteria. The random sample drawn from the first selected cohort for questionnaire dispatch comprised 3,000 persons, of whom 2,268 were contacted (732 were under supervision and/or had a postal address abroad). The second cohort (only tilidine/naloxone) comprised 1,000 individuals, of whom 769 were contacted (231 were under supervision and/or had a postal address abroad). After the first dispatch, 557 questionnaires with signed consent were returned, after the second dispatch, a total of 730 were returned, resulting in a response rate of 23.7%. Of these questionnaires, 58 were excluded due to missing responses to question 1 or no arthrosis and/or back pain. Additionally, two questionnaires were excluded because of incomplete responses, and nine individuals were excluded as a distinct pseudonymization was not possible. The final sample consisted of 661 individuals, leading to a net response rate of 21.8%.

Compared to the contacted random sample (*n* = 3,037), responders were younger (mean age 69.4 years SD 12.5 vs. 71.3 SD 13.8) and slightly more often female (75.5% vs. 74.38%). One-third of the included persons reported back pain, 14% arthrosis pain, and just over half of the individuals reported both types of pain. A descriptive presentation regarding results of the survey instruments used and other variables used for the subgroup analysis can be found in Additional file 4.

**Fig. 2:**
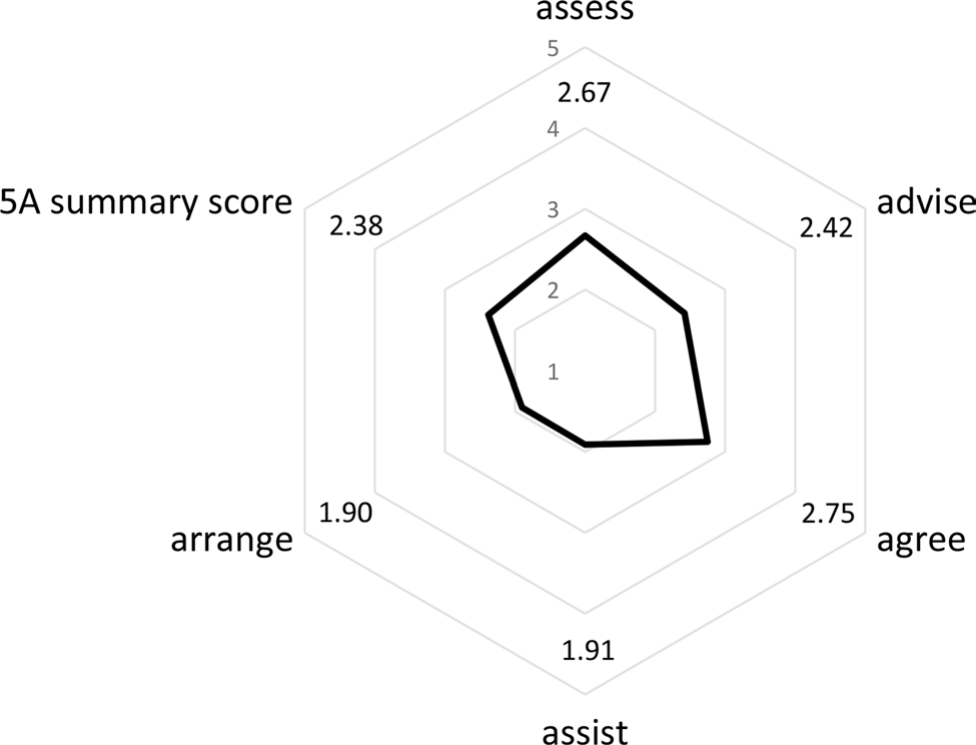
Mean values of the 5A model of the Patient Assessment of Chronic Illness Care (*n* = 661)

The assessment of the quality of healthcare according to the CCM, described by the PACIC-5A (sub)scores, is presented descriptively in Fig. 2. With a possible score of 1 (“almost never”) to 5 (“almost always”), the subscale regarding the follow up of the treatment plan “arrange” had the lowest rating with a mean of 1.9, the subscale about joint treatment planning “agree” was rated best with a mean of 2.75.

Internal consistency calculated by Cronbach’s α ranged from an acceptable 0.72 for “arrange” to a good 0.94 for the “5A Summary Score”, with a declined Cronbach’s α of 0.66 for “advise” (behavior change advise/information about personal health harms and benefits) (see Table 2). Floor effects occurred more clearly than ceiling effects. The subscales “assist” (support for behavior change) and “arrange”, which were rated lowest overall, showed pronounced floor effects (23.5%/23.4% of persons with subscale score 1).

**Table 2:** Descriptive evaluation and internal reliability of the patient assessment of chronic illness care (*n* = 661)

	*n*	missing	mean	SD	Cronbach’s α	floor effect	ceiling effect
assess	584	77	2.67	1.13	0.82	8.9%	2.2%
advise	573	88	2.42	0.86	0.66	4.2%	0.5%
agree	584	77	2.75	1.13	0.85	8.2%	3.1%
assist	582	79	1.91	0.87	0.75	23.5%	0.5%
arrange	578	83	1.90	0.85	0.72	23.4%	0.3%
5A Summary Score	552	109	2.38	0.86	0.94	1.3%	0%

**Fig. 3:**
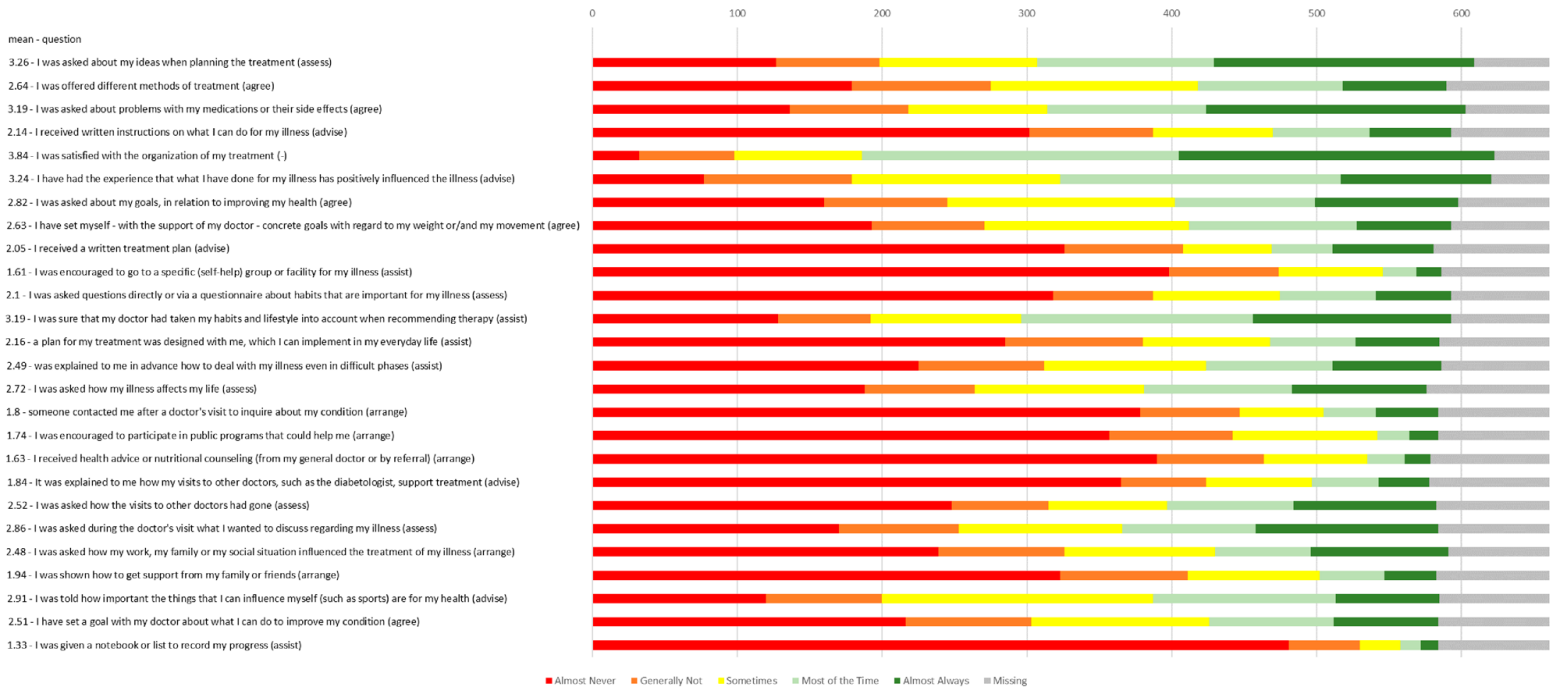
Mean values and frequency distribution of the 26 items of the PACIC questionnaire (*n* = 661)

In addition, Fig. 3 provides a detailed overview of the number of patients answering, the frequency distribution of responses and mean scores for the 26 individual questions. The mean scores of the individual questions showed a larger range than the summary scores of the PACIC-5A evaluation, with a minimum of 1.33 (giving list/booklet to record progress) to a maximum of 3.84 (satisfaction with organization of treatment).The subgroup sizes for the analyses described below, related to the PACIC-5A mean values, are detailed in Additional file 5. A clear association of quality of healthcare as specified by the CCM and patient characteristics could not be found. Subgroup analyses for the independent variables sex, migration background, and pain diagnosis (see Additional file 6) showed no statistically significant group differences. Table 3 illustrates a deterioration in mean PACIC-5A scores with increasing age, although a slight improvement was initially observed between the age group 18–49 and 50–69 except for the “assess” subscale. Only the scales “assess”, “agree”, and “5A summary score” showed statistically significant group differences. Finally, concerning the level of education, mean PACIC-5A scores were higher in individuals with a higher degree, with a consistent maximum for a technical school degree and a slightly decreasing mean score again for persons with a university degree. The group differences for the level of education in the scales “assess”, “agree”, and “5A summary score” showed the strongest effect sizes, followed by “advise”; all four of them presenting with statistically significant group differences.

The quality of healthcare as specified by the CCM was associated with the two variables concerning patient’s health situation “psychological distress” and “symptoms of opioid Substance Use Disorder”. First, lower quality of healthcare according to the CCM was observed in persons with higher psychological distress (see Table 3). This decrease in mean PACIC-5A values was more pronounced in the group of the most highly distressed individuals; statistical significance was reached for the subscales “advise”, “agree”, and “5A summary score”. Second, there was a slight tendency toward lower mean PACIC-5A scores with more pronounced symptoms of opioid Substance Use Disorder; group differences reached statistical significance for the “advise” subscale only. Apart from this, the assessment of the intensity of pain and the related impairment measured by the GCPS showed no clear trend regarding a correlation with the patient’s assessment of the quality of healthcare according to the CCM. Only the subscale rating about behavioral counseling (“advise”) showed a statistically significant weak negative correlation with a higher GCPS level.

**Table 3:** Subgroup analyses of the patient assessment of chronic illness care on patient characteristics and patient’s health situation

independent variables	value	assess		advise		agree		assist		arrange		5A summary score	
		X̅	effect size^a^	X̅	effect size^a^	X̅	effect size^a^	X̅	effect size^a^	X̅	effect size^a^	X̅	effect size^a^
**patient characteristics**													
age	18–49	2.94	-0.125^*^	2.39	-0.035	2.79	-0.094^*^	1.89	-0.031	1.89	-0.034	2.43	-0.076^*^
	50–69	2.78		2.45		2.86		1.95		1.94		2.44	
	70–89	2.57		2.40		2.67		1.89		1.87		2.32	
	≥ 90	2.14		2.21		2.16		1.83		1.83		2.13	
highest educational qualification	currently at school/vocational training	-	0.188^*^	-	0.143^*^	-	0.187^*^	-	0.104	-	0.097	-	0.170^*^
	no degree	2.07		2.10		2.06		1.59		1.59		1.92	
	vocational training/apprenticeship	2.60		2.36		2.67		1.87		1.87		2.32	
	technical school	3.15		2.78		3.24		2.24		2.12		2.73	
	university degree	2.94		2.52		2.94		1.92		1.93		2.52	
**patient’s health situation**													
psychological distress - severity	none	2.80	-0.069	2.58	-0.176^*^	2.87	-0.096^*^	1.94	-0.033	1.93	-0.030	2.49	-0.120^*^
	low	2.71		2.45		2.74		1.93		1.93		2.39	
	moderate	2.70		2.38		2.75		1.98		1.95		2.40	
	severe	2.45		2.01		2.49		1.69		1.70		2.08	
symptoms of opioid Substance Use Disorder	none	2.71	-0.038	2.50	-0.109^*^	2.81	-0.052	1.95	-0.020	1.94	-0.022	2.43	-0.064
	low	2.71		2.40		2.73		1.91		1.92		2.36	
	moderate	2.63		2.33		2.82		1.86		1.84		2.37	
	severe	2.40		2.17		2.45		1.71		1.71		2.13	
intensity of pain-related impairment	no pain	2.73	0.054	2.68	-0.089^*^	2.89	-0.025	1.81	0.086	1.81	0.087	2.44	0.009
	low pain and low pain-related- impairment	2.88		2.52		2.94		1.98		1.98		2.51	
	severely pain and low pain-related impairment	2.53		2.50		2.66		1.88		1.85		2.32	
	severely pain-related-impairment, moderate limiting	2.63		2.46		2.82		1.82		1.80		2.39	
	severely pain-related-impairment, severely limiting	2.77		2.36		2.76		1.97		1.96		2.40	

The mean subscores can only be calculated based on non-missing values for both the independent variable and the respective PACIC-5A score for an individual, which may lead to different subgroup sizes

Test for statistical significance and calculation of the effect measure for the categorical variables “age group”, “psychological distress – severity”, “expression of an addiction problem” with the metric values underlying the categorization

^*^p < 0.05 (test for statistical significance of group differences: Spearman-Rho)

X̅ Mean score of the PACIC-5A (sub)scale

^a^ Spearman’s Rank correlation coefficient

The association of pain treatment aspects with healthcare quality as specified by the CCM was examined in subgroup analysis. Positive correlations can be observed across all aspects and PACIC-5A scales with statistically significant group differences. First, in the area of specialized outpatient pain therapy, statistically significant but, evaluated by effect measure, rather weak effects could be shown, with the highest effect measure in the area of the assessment of behavioral health risks (“assess”) with an Eta of 0.198 and an Eta of 0.141 and above for the other scales (see Table 4). Regarding the guideline [6] elements “setting of therapy goals” and “comprehensive treatment concept”, there were five cases of overlap in content between a subscale of the PACIC-5A and the guideline-compliant aspect. Therefore, the results of these analyses are indicated accordingly in Table 4, and are not considered further in the analysis. The strongest correlations were found for the variable regarding a comprehensive treatment concept with a rounded Eta value for subscales of 0.4. Furthermore, the subgroup analysis concerning the setting of therapy goals showed weaker correlation, with the strongest correlation with the PACIC-5A subscales “assess” (Eta 0.314) and “advise” (Eta 0.291).

**Table 4:** Subgroup analyses of the patient assessment of chronic illness care on pain treatment aspects

independet variable	value	assess		advise		agree		assist		arrange		5A summary score	
		X̅	Eta	X̅	Eta	X̅	Eta	X̅	Eta	X̅	Eta	X̅	Eta
outpatient pain therapy	yes	2.86	0.198^*^	2.52	0.141^*^	2.90	0.153^*^	2.02	0.144^*^	2.01	0.148^*^	2.51	0.177^*^
	no	2.41		2.27		2.55		1.76		1.75		2.20	
setting of therapy goals	yes	2.92	0.314^*^	2.58	0.291^*^	*2.99* ^†^	*0.311* ^*†^	2.05	0.211^*^	2.03	0.210^*^	2.58	0.328^*^
	no	2.14		2.03		*2.21* ^†^		1.64		1.64		1.96	
comprehensive treatment concept	yes	3.39	0.419^*^	2.96	0.425^*^	*3.55* ^†^	*0.471* ^*†^	*2.46* ^*†*^	*0.422* ^*†^	*2.41* ^*†*^	*0.414* ^*†^	*3.02* ^*†*^	*0.500* ^*†^
	no (none/not comprehensive)	2.34		2.17		*2.38* ^†^		*1.66* ^*†*^		*1.65* ^*†*^		*2.08* ^*†*^	

* *p* < 0.05 (test for statistical significance of group differences: Mann-Whitney U test)

†Content overlap between subscales of the PACIC-5A and the independent variables

Table 5 presents the subgroup analyses for the categorized interdisciplinary pain management procedures which might be a guideline [6] compliant pain treatment aspects in healthcare (please refer to Fig. 1). Across all procedures and scales, an improvement in PACIC-5A mean values was observed when procedures were performed. This tendency was present but least pronounced in the subscale regarding individual assistance for behavior change (“assist”) followed by the subscale concerning the follow up of treatment plans (“arrange”). Regarding the procedure, psychotherapy showed in terms of effect size (Eta) the strongest correlation with PACIC-5A scores, followed by special medical pain management procedures. Finally, the number of categories of procedures of interdisciplinary pain therapy performed in patient’s pain therapy was considered. There was a positive correlation between better quality of healthcare in accordance with the CCM and an increasing number of categories utilized. This effect is most pronounced in the subscale “assess” (Eta 0.192), followed by “agree” (Eta 0.165) and “advise” (Eta 0.149).

**Table 5:** Subgroup analysis of the patient assessment of chronic illness care for interdisciplinary pain therapy

categorized procedures of interdisciplinary pain therapy	value	assess		advise		agree		assist		arrange		5A summary score	
		X̅	effect size	X̅	effect size	X̅	effect size	X̅	effect size	X̅	effect size	X̅	effect size
special medicinal pain management procedures	yes	2.78	0.138^*a^	2.46	0.091^*a^	2.84	0.116^*a^	1.95	0.068^a^	1.94	0.077^*a^	2.44	0.117^*a^
	no	2.43		2.29		2.55		1.82		1.80		2.22	
remedies	yes	2.73	0.135^*a^	2.43	0.065^*a^	2.79	0.106^*a^	1.91	0.001^a^	1.90	0.018^a^	2.41	0.103^*a^
	no	2.23		2.25		2.40		1.91		1.86		2.11	
psychotherapy	yes	2.93	0.164^*a^	2.57	0.134^*a^	3.00	0.163^*a^	2.08	0.144^*a^	2.04	0.124^*a^	2.56	0.156^*a^
	no	2.54		2.33		2.62		1.82		1.82		2.28	
day patient/inpatient procedures	yes	2.74	0.096^*a^	2.47	0.106^*a^	2.83	0.103^*a^	1.93	0.043^a^	1.93	0.060^*a^	2.43	0.096^*a^
	no	2.51		2.28		2.57		1.85		1.82		2.25	
other non-medicinal complementary procedures	yes	2.78	0.117^*a^	2.47	0.090^*a^	2.83	0.095^*a^	1.94	0.051^a^	1.94	0.067^a^	2.43	0.089^*a^
	no	2.50		2.32		2.61		1.85		1.83		2.28	
number of categories of procedures of interdisciplinary pain therapy	0	2.01	0.192^*b^	2.10	0.149^*b^	2.01	0.165^*b^	1.62	0.119^*b^	1.60	0.125^*b^	1.90	0.172^*b^
	1	2.39		2.14		2.50		1.88		1.82		2.20	
	2	2.64		2.48		2.75		1.82		1.84		2.35	
	3	2.63		2.35		2.74		1.95		1.93		2.35	
	4	2.67		2.43		2.76		1.84		1.85		2.37	
	5	3.01		2.59		3.03		2.10		2.07		2.60	

* *p* < 0.05 (test for statistical significance of group differences: Mann-Whitney U test (single procedure), Spearman-Rho (number of categories))

a Eta

b Spearman’s Rank correlation coefficient

## Discussion

The cross-sectional survey cohort comprised 661 individuals insured by “DAK-Gesundheit” with long-term opioid therapy and chronic back and/or arthrosis pain. The aim of this analysis was to analyze quality of outpatient healthcare according to the CCM from the patient’s perspective. Survey participants rated the quality of healthcare according to the CCM rather low. Patient characteristics showed minimal to no influence on PACIC-5A scores, while patient’s health situation demonstrated only partial correlations, including a negative correlation with psychological distress. Conversely, there was a consistently positive correlation observed with the delivery of aspects of pain treatment, such as the setting of therapy goals and a comprehensive treatment concept. Selection criteria resulted in 0.7% (first selection) and 0.6% (second selection) of insured adults receiving long-term opioid therapy and chronic back and/or arthrosis pain.

### PACIC-5A

As initially assumed, the cohort under consideration predominantly (85%) exhibited pain related high disability, moderately to severely limiting according to the GCPS. Therefore, the utilization of the PACIC-5A questionnaire in this cohort of highly burdened chronically ill individuals can be deemed appropriate. The psychometric quality of the PACIC-5A assessment, as measured by Cronbach’s α, demonstrated acceptable to good internal consistency, with the exception of the “advise” subscale, which narrowly missed the threshold of 0.7 for acceptable internal consistency (Cronbach’s α = 0.66).

In terms of the overall evaluation of healthcare quality according to the CCM, the mean subscale values were lowest for the subscales on follow-up of treatment plans “arrange” (1.90) and on aid for patients in achieving set goals “assist” (1.91). Furthermore, pronounced floor effects for these two subscales suggest that these aspects of healthcare according to the CCM were entirely absent for almost a quarter of respondents. The two aforementioned aspects represent crucial pillars of healthcare according to the CCM, thus offering significant potential for enhancing the quality of healthcare for chronic pain patients with long-term opioid therapy. Though, these results need to be interpreted in regards of the German healthcare system focusing on medical treatment. Therefore, preventive and activating aspects outlined in the CCM might not be met by health services due to personnel, organizational, and financial constraints. In these terms low sores in PACIC-5A subscales need to be addressed by health policy especially since they cover aspects recommended by the LONTS guideline [6]. The improvement of the “assist” aspect could involve encouraging of patients to join (self-help) groups or facilities for their illness. To enhance the rating of the “arrange” subscale, regular provision of health advice and nutritional counseling could be implemented. Patients could also be encouraged to participate in public programs and contacted more frequently between appointments to check on their condition. Additionally, they could be educated on how to seek support from family and friends. Furthermore, while the “advise” (2.42), “assess” (2.67), and “agree” (2.75) subscales were rated higher in mean values within a possible range of PACIC-5A rating from 1 to 5, there is still the possibility of improvement toward optimal healthcare according to the CCM. Specifically, the “advise” subscale could be improved by providing patients with better information on the support of their treatment by other specialist physician visits.

To our knowledge, this is the first time that the PACIC-5A survey has been conducted in a German cohort of patients experiencing chronic back and/or arthrosis pain and receiving long-term opioid therapy. Several studies examined the quality of outpatient healthcare according to the CCM using the PACIC-5A in patients with different medical indications in Germany. These studies mirrored our survey’s findings, with the “assist” and “arrange” scales consistently showing the lowest values [27–30]. A survey conducted on patients after an acute inpatient stay due to colorectal carcinoma (*n* = 816) evaluated similar values, with the present survey rating the subscale “advise“ 0.3 points lower and “assess“ 0.3 points higher [27]. Patients surveyed on the quality of outpatient healthcare in accordance with the CCM after an acute inpatient stay for mental disorders (n = 2,289), showed an average of 0.4 points higher result across all scores. Individual assistance (“assist”) in particular deviated downward by -0.7 points in the present cohort [28], which is one of the lowest-rated subscales in the current study. A PACIC-5A survey among persons with osteoarthritis (n = 1021) also showed higher scores across all (sub)scales of the PACIC-5A compared to the present analysis [29]. A further survey in a cohort of individuals with obesity (n = 117) described scores of comparable magnitude to the present survey [30].

### Patient characteristics

We assumed that patient characteristics could possibly influence healthcare quality as specified by the CCM. However, our results indicate minimal to no correlations. A younger age and a higher educational level showed weak positive, partly statistically significant correlation with healthcare quality ratings according to the CCM. This trend regarding age [28, 29] and educational level [29] also appeared in literature. The independence of the PACIC-5A scores from sex in terms of statistical significance was also shown in other surveys [21, 28, 29]. As it pertains to aspects of equity in health, patient characteristics should not influence the quality of healthcare according to the CCM [31]. For migration background, pain diagnosis, and sex, no deviation from this normative principle could be shown in our analysis. However, older and less educated individuals appear to exhibit a slight tendency towards poorer-rated healthcare quality in terms of the CCM. Patient-centered healthcare, with the integration of shared decision-making, is to be understood as a process that requires productive communication between physician and patient. This communication is influenced by patient preferences and physicians’ competencies to communicate in a manner appropriate to the individual target group. Furthermore, even if we assumed that patient characteristics act as independent variables, it cannot be excluded that patients valued treatment differently based on their characteristics. Future research should investigate the extent to which certain aspects of healthcare according to the CCM are not offered, for example, to older and less educated individuals, or are not preferred by them. Recommendations for physician-patient communication tailored to subgroups should be developed.

### Patient’s health situation

The second group of variables, patient’s health situation, assumed to be influenced by healthcare quality according to the CCM, correlated only partially. A higher degree of anxiety and depression symptoms, as well as a higher degree of opioid addiction problems, tended to correlate slightly with a poorer quality of healthcare as specified by the CCM. Similarly, another evaluation of the PACIC-5A score found an association between higher depressive severity levels on the PHQ-9 instrument and lower PACIC-5A scores [29]. This trend may indicate a protective effect of good quality healthcare according to CCM on risks commonly associated with chronic pain or opioid treatment, such as psychological distress, particularly depression [32], or opioid addiction [6]. It should be noted that the observed effects are small, and causal relationship cannot be verified by this cross-sectional survey. Furthermore, the GCPS [24] is a crucial variable for assessing the treatment success of long-term opioid therapy. The corresponding subgroup analysis revealed only one statistically significant correlation between healthcare quality according to the CCM and the insured individuals’ pain experience. The lack of a clear trend might be attributed to many possible causes: results of the GCPS show strong ceiling effects as across the 5 levels of the GCPS, 85% are distributed to the two highest classifications. This indicates long-term opioid therapy to be prescribed to a highly burdened patient group suffering from pain for a long time. Therefore, a more sensitive instrument than the GCPS would be desirable. Lastly, the causal relationship of a change in healthcare quality as specified by the CCM on the patient’s health situation cannot be demonstrated in a cross-sectional design. Therefore, future longitudinal studies should investigate the influence of the quality of healthcare according to the CCM, particularly on pain intensity and pain-related impairment, in greater detail.

### Pain treatment aspects

The last category of subgroup analysis, pain treatment aspects, revealed the strongest correlations with the assessment of healthcare quality according to the CCM, particularly in the domain of guideline-compliant [6] pain treatment aspects, namely therapy goals and treatment concept. Although there was partial content overlap between PACIC-5A subscores and these variables, the non-overlapping subscales also exhibiting the effect in the same direction. Given that only a third of respondents in our analysis reported a comprehensive treatment concept, this area appears to offer great potential for enhancing the quality of healthcare as specified by the CCM. It should be noted that the subjective perception of patients to the setting of therapy goals and the development of a comprehensive therapy concept may differ from the objective implementation in healthcare. However, since concepts need to be remembered by patients to facilitate a behavioral change, this subjectivity does not appear to be disadvantageous. Additionally, the implementation of different procedures of an interdisciplinary pain therapy, such as psychotherapy, remedies or complementary procedures, which can form part of a comprehensive treatment concept, also demonstrated a positive correlation with PACIC-5A scores. Hence, health participation in diverse procedures is associated with a higher healthcare quality according to the CCM. Moreover, the slightly higher rating of the healthcare quality as specified by the CCM among insured individuals who received outpatient pain therapy treatment could be attributed to the specialized expertise of the providers [33]. Specialized pain physicians in Germany can bill more time-intensive consultations per patient through SHI, potentially resulting in increased resources for healthcare in accordance with the CCM. It can be concluded that treatment according to the guideline [6] recommendations constitutes an important contribution to the quality assurance of outpatient pain management according to the CCM. Following the assumption of a bidirectional relationship, the guideline [6] recommendations seem to align with the CCM, and aspects of treating chronically ill persons seem to be addressed correctly in the guideline [6]. Therefore, it seems sensible to promote guideline [6] adherence, such as increasing physician awareness of the guideline [6] and improving communication with patients about the importance of multimodal therapy. Pain therapists also appear to play an important role in delivering high-quality treatment according to the CCM. However, there still appears to be potential for improvement in the quality of healthcare as specified by the CCM provided by pain therapists.

### Chronic care model in Germany

Developed as an ideal healthcare model for chronically ill patients, the CCM aims to promote behavioral changes and optimize disease management. However, implementing the CCM in the German healthcare system is challenging and seems more feasible in settings with sufficient resources, such as specialized pain therapy. The root of the problem is not just with individual physicians but also at multiple levels, such as the current healthcare system structure, where time resources for each patient are often severely limited. Additionally, some of the aspects gathered by the PACIC-5A questionnaire are not covered by the SHI system and, as such, are ineligible for reimbursement. This might be the reason, why our results seem to be in line with other German studies on other indications. Nevertheless, guidelines recommend this several aspects of the CCM perceiving pain in regards of the biopsychosocial disease model [6].

### External validity

The survey is representative for the German SHI “DAK-Gesundheit”. Compared to the entire SHI population in Germany (which covers 88% of the population [34]), “DAK-Gesundheit” has a slightly older population with a higher proportion of women. Since the benefit package, contractual relations between health insurers and providers and other legal requirements for all insured persons in the SHI population are essentially the same, it can be assumed that the results can be extrapolated to the entire population of insured persons. Due to the slight shift in the structure of the insured, it can be presumed that the rating of the PACIC-5A might be slightly better in the entire SHI population, as the female gender and a higher age tended to be associated with poorer ratings of the PACIC-5A.

### Limitations

The current analysis offers valuable insights into outpatient healthcare quality according to the CCM for patients with chronic back and/or arthrosis pain who received long-term opioid therapy. However, some limitations must be considered. The bivariate analysis only establishes correlation, not causality, and potential possible confounding variables are not accounted for. Additionally, the assumed direction of effects cannot be definitively determined. The cross-sectional design precludes the demonstration of long-term effects of the level of healthcare quality as specified by the CCM, such as on the GCPS. Individuals with the same quality of healthcare may have responded differently based on their level of education. Conversely, it is possible that patients are treated differently by physicians depending on their education level, or that patients themselves approach medical treatment with varying expectations. Moreover, while the ultra-brief PHQ-4 questionnaire, consisting of 4 items, is a validated instrument [17], some loss of information in measuring psychological distress is conceivable compared to the more detailed PHQ-9 questionnaire. Additionally, the survey results may be affected by recall bias due to the survey period. Though most of the question addressed health services during the last 3 to 12 months. Furthermore, it remains unknown whether all respondents fully understood the questions. Finally, a net response rate of 21.8% suggests the possibility of a non-response bias; however, non-response analysis revealed a comparable age and gender distribution.

## Conclusions

The evaluation of quality of outpatient healthcare, according to the CCM, from the perspective of the insured among a small sample of persons with long-term opioid therapy and chronic back and/or arthrosis pain showed a positive correlation with healthcare aspects according to the German guideline recommendations [6]. Currently, there still seems to be potential for improvement of the quality of healthcare as specified by the CCM, so that it should be examined to what extent the implementation of outpatient healthcare for chronic pain can be refined in accordance with the CCM, e.g., by enhancing guideline-compliant [6] outpatient healthcare. A comprehensive treatment concept could be crucial for providing high-quality healthcare to chronically ill individuals, particularly within a functionally differentiated healthcare system like that of Germany. The CCM is an ideal model for healthcare in chronic conditions, which seems difficult to be fully implement in Germany. Various reasons at the micro, meso and macro levels of the health care system might contribute to these difficulties, though their mechanism of action for the health care of chronic patients needs further research. Furthermore, we could not detect a correlation between the patient’s pain situation and healthcare quality according to CCM. This topic, which is crucial for the health situation of chronic pain patients, should be investigated in more detail by means of a longitudinal study design using a sensitive measurement instrument.

## Electronic supplementary material

Below is the link to the electronic supplementary material.

Supplementary Material 1: Excerpt of questions relevant to the present analysis of the patient questionnaire from the insured person survey (translated from German).

Supplementary Material 2: Description of the variables used in the analysis.

Supplementary Material 3: Assignment systematics of the questions of the epidemiological addiction survey 2015 to the DSM-V criteria and description of the evaluation systematics.

Supplementary Material 4: Descriptive results of the independent variables used in subgroup analysis.

Supplementary Material 5: Subgroup sizes of mean value calculation of the Patient Assessment of Chronic Illness Care (PACIC-5A) subscores.

Supplementary Material 6: Subgroup analysis of the scores of the 5A model of the Patient Assessment of Chronic Illness Care (PACIC-5A) on migration background, sex and pain diagnosis.

## Data Availability

The datasets generated and analyzed during the current study are not publicly available due to the restriction in data transfer stated in the informed consent form signed by participants.

## Abbreviations

ATC: Anatomical Therapeutic Chemical Classification System
CCM: Chronic Care Model
DSM-V: Diagnostic and Statistical Manual of Mental Disorders, Fifth Edition
GCPS: Graded Chronic Pain Scale
ICD-10-GM: International Classification of Diseases – 10th Revision – German Modification
LONTS: Langzeitanwendung von Opioiden bei chronischen nicht-tumorbedingten Schmerzen (evidence-based guideline for the long-term opioid therapy for chronic non-cancer-related pain)
PACIC: Patient Assessment of Chronic Illness Care
PACIC-5A: 5A model of the Patient Assessment of Chronic Illness Care
PHQ-4: Patient Health Questionnaire-4
SD: Standard deviation
SHI: Statutory health insurance
