# Supplementary material for: Patient-reported quality of outpatient healthcare in patients with chronic back or arthrosis pain with long-term opioid therapy in Germany
Source: BMC Prim Care. 2025 Jun 21;26:200. doi: 10.1186/s12875-025-02881-3 (PMC12181890; doi:10.1186/s12875-025-02881-3)
Supplement: Supplementary file 1 — Supplementary Material 1: Excerpt of questions relevant to the present analysis of the patient questionnaire from the insured person survey (translated from German). [file 12875_2025_2881_MOESM1_ESM.docx]

**Additional file 1:** Excerpt of questions relevant to the present analysis of the patient questionnaire from the insured person survey (translated from German).

1. **First of all, we are interested in your pain situation. In the last year (2020), have you had severe pain for at least three months? If yes, which ones?**

|  | | **No,** I have **not** had **severe pain in the** past year | | | **🡪 If no, please do not fill in the questionnaire any further** | | | | |
| --- | --- | --- | --- | --- | --- | --- | --- | --- | --- |
|  | | **Yes** ***(multiple answers are possible.)*** | |  | |  |  |  |  |
|  | | severe **back pain** | | | |  | |  |  |
|  | | severe **arthritis pain (joint pain)** | | | | |  | | |
|  | | severe **other pain** | | | | | |  | |

1. **Indicate the severity of your pain below. Please tick on the scales below how much pain you feel (under your usual medication).**

**A value of 0 means you do not have any pain, a value of 10 means you are suffering from pain that you cannot imagine to be stronger. The numbers in between indicate gradations of pain intensity.**

1. **First, please state your current pain level:**

| 0 | 1 | 2 | 3 | 4 | 5 | 6 | 7 | 8 | 9 | 10 |
| --- | --- | --- | --- | --- | --- | --- | --- | --- | --- | --- |

| No  Pain |  | Strongest  imaginable pain |
| --- | --- | --- |

1. **Now please state your average pain intensity during the last four weeks:**

| 0 | 1 | 2 | 3 | 4 | 5 | 6 | 7 | 8 | 9 | 10 |
| --- | --- | --- | --- | --- | --- | --- | --- | --- | --- | --- |

| No  Pain |  | Strongest  imaginable pain |
| --- | --- | --- |

1. **Now please state your greatest pain intensity during the last four weeks:**

| 0 | 1 | 2 | 3 | 4 | 5 | 6 | 7 | 8 | 9 | 10 |
| --- | --- | --- | --- | --- | --- | --- | --- | --- | --- | --- |

| no  Pain |  | strongest  imaginable pain |
| --- | --- | --- |

1. **Now indicate what level of pain would be bearable for you if the treatment was successful:**

| 0 | 1 | 2 | 3 | 4 | 5 | 6 | 7 | 8 | 9 | 10 |
| --- | --- | --- | --- | --- | --- | --- | --- | --- | --- | --- |

| no  Pain |  | strongest  imaginable pain |
| --- | --- | --- |

1. **The following questions are about your pain during the last three months. For this period, we would like to know more about the effects of the pain.**

1. **In the last three months, how many days were you unable to do your usual activities (e.g. work, school, household) because of pain?**

*Please tick the numbers that apply.*

| On about $\begin{matrix} ⓪ & ⓪ \\ ① & ① \\ ② & ② \\ ③ & ③ \\ ④ & ④ \\ ⑤ & ⑤ \\ ⑥ & ⑥ \\ ⑦ & ⑦ \\ ⑧ & ⑧ \\ ⑨ & ⑨ \end{matrix}$ days | **Completion aid** | |
| --- | --- | --- |
|  | Please tick the two numbers that make up the number of days that applies to you.  **Examples:**  1. For the answer “on about **three** days”, tick **0 in the left column** and **3 in the right column.**  2. If you answer “about **twelve** days”**,** tick **1 in the left column** and tick **2 in the right column.** | **Example of the answer**  **“on about twelve days”:**  On about $\begin{matrix} ⓪ & ⓪ \\ ① & ① \\ ② & ② \\ ③ & ③ \\ ④ & ④ \\ ⑤ & ⑤ \\ ⑥ & ⑥ \\ ⑦ & ⑦ \\ ⑧ & ⑧ \\ ⑨ & ⑨ \end{matrix}$ days 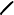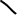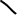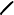 |

In the **following pain impairment rating,** a value of 0 means you do not have any impairment. A value of 10 means you are completely impaired. The numbers in between indicate gradations of impairment.

1. **To what extent has the pain affected your daily life (dressing, washing, eating, shopping, etc.) in the last three months?**

| 0 | 1 | 2 | 3 | 4 | 5 | 6 | 7 | 8 | 9 | 10 |
| --- | --- | --- | --- | --- | --- | --- | --- | --- | --- | --- |

| No  impairment |  | Complete  impairment |
| --- | --- | --- |

1. **To what extent has the pain interfered with your leisure activities or activities with family or friends in the last three months?**

| 0 | 1 | 2 | 3 | 4 | 5 | 6 | 7 | 8 | 9 | 10 |
| --- | --- | --- | --- | --- | --- | --- | --- | --- | --- | --- |

| No  impairment |  | Complete  impairment |
| --- | --- | --- |

1. **To what extent has the pain affected your ability to work (including housework) in the last three months?**

| 0 | 1 | 2 | 3 | 4 | 5 | 6 | 7 | 8 | 9 | 10 |
| --- | --- | --- | --- | --- | --- | --- | --- | --- | --- | --- |

| No  impairment |  | Complete  impairment |
| --- | --- | --- |

Graded Chronic Pain Scale Items and Scoring (GCPS, von Korff et al. 1992) Public licence (CC) BY-NC-SA 4.0 Response format of question 9 was modified.

1. **Thinking back to your course of disease, by whom have you been examined or treated for your pain so far? Please indicate also when you have had your last contact (counselling or treatment) with a representative of the respective professional group because of the pain.**

- **Please make a cross *in each line.***

| **Professional groups** | **Never**  **before** | **In the last three months** | | **In the last four to twelve months** | **More than a year ago** |
| --- | --- | --- | --- | --- | --- |
| Family doctor^[[1]](#footnote-1)^ |  |  | |  |  |
| Surgeon |  |  |  | |  |
| Internist |  |  |  | |  |
| Neurosurgeon |  |  |  | |  |
| Neurologist |  |  |  | |  |
| Orthopaedist/ trauma surgeon |  |  |  | |  |
| Physiotherapist |  |  |  | |  |
| Psychiatrist |  |  |  | |  |
| Psychotherapist |  |  |  | |  |
| Radiologist |  |  |  | |  |
| Pain therapist |  |  |  | |  |
| Alternative practitioner |  |  |  | |  |
| Other:_________________ |  |  |  | |  |

1. **There are very different treatment options for pain. Please indicate which procedures you have tried so far and in which period of time this treatment took place?**

Please tick which of the treatment measures listed below you have received.

- **Please make a cross *in each line.***

|  | **When did you receive the last treatment?** | | | |
| --- | --- | --- | --- | --- |
|  | **Never before** | **In the last three months** | **Four to twelve months ago** | **More than a year ago** |
| Non-opioid painkillers (e.g. paracetamol, ibuprofen, ASS, diclofenac, naproxen, metamizole) |  |  |  |  |
| Opioid painkillers (e.g. fentanyl, tilidine, hydromorphone, oxycodone, tapentadol, buprenorphine, tramadol, morphine) |  |  |  |  |
| Infusions |  |  |  |  |
| Injections into the pain area, nerve blocks |  |  |  |  |
| Spinal cord injections (e.g. epidural) |  |  |  |  |
| Spinal cord probe (SCS) or pump systems |  |  |  |  |
| Physiotherapy |  |  |  |  |
| Manual therapy |  |  |  |  |
| Massages, baths, cold/heat therapy |  |  |  |  |
| Electrical nerve stimulation (TENS) |  |  |  |  |
| Acupuncture |  |  |  |  |
| Chiropractic |  |  |  |  |
| Psychotherapy |  |  |  |  |
| Health resort/rehab treatment |  |  |  |  |
| Outpatient/partial inpatient/inpatient pain therapy |  |  |  |  |
| Day clinic |  |  |  |  |
| Other:_____________________ |  |  |  |  |

1. **During the last two weeks, how often did you feel affected by the following complaints?**

- **Please make a cross *in each line.***

|  | Not at all | On individual days | On more than half of the days | Almost every day |
| --- | --- | --- | --- | --- |
| Little interest or pleasure in doing things |  |  |  |  |
| Feeling down, depressed or hopeless |  |  |  |  |
| Feeling nervous, anxious or on edge |  |  |  |  |
| Not being able to stop or control worrying |  |  |  |  |

1. **Think about opioid therapy for medical treatment you have received over the past 6 months due to persistent pain. (If it has been more than 6 months since you have seen your attending doctor, think about your most recent visit.)**

- **Please make a cross in each line.**

| **Over the past 6 months, I was:** | | **Almost never** | **Usually not** | **Sometimes** | **Most of the time** | **Almost always** |
| --- | --- | --- | --- | --- | --- | --- |
| 1. | Asked for my ideas when we made a treatment plan. |  |  |  |  |  |
| 2. | Given choices about treatment to think about. |  |  |  |  |  |
| 3. | Asked to talk about any problems with my medicines or their effects. |  |  |  |  |  |
| 4. | Given a written list of things I should do to improve my health. |  |  |  |  |  |
| 5. | Satisfied that my care was well organized. |  |  |  |  |  |
| 6. | Shown how what I did to take care of my illness influenced my condition. |  |  |  |  |  |
| 7. | Asked to talk about my goals in caring for my illness. |  |  |  |  |  |
| 8. | Helped to set specific goals to improve my eating or exercise. |  |  |  |  |  |
| 9. | Given a copy of my treatment plan. |  |  |  |  |  |
| 10. | Encouraged to go to a specific group or class to help me cope with my chronic illness. |  |  |  |  |  |
| 11. | Asked questions, either directly or on a survey, about my health habits. |  |  |  |  |  |
| 12. | Sure that my doctor or nurse thought about my values and my traditions when they recommended treatments to me. |  |  |  |  |  |
| 13. | Helped to make a treatment plan that I could do in my daily life. |  |  |  |  |  |
| 14. | Helped to plan ahead so I could take care of my illness even in hard times. |  |  |  |  |  |
| 15. | Asked how my chronic illness affects my life. |  |  |  |  |  |
| 16. | Contacted after a visit to see how things were going. |  |  |  |  |  |
| 17. | Encouraged to attend programs in the community that could help me. |  |  |  |  |  |
| 18. | Referred to a dietitian, health educator, or counselor. |  |  |  |  |  |
| 19. | Told how my visits with other types of doctors, like the diabetologist, helped my treatment. |  |  |  |  |  |
| 20. | Asked how my visits with other doctors were going. |  |  |  |  |  |
| 21. | Asked what I would like to discuss about my illness at that visit. |  |  |  |  |  |
| 22. | Asked how my work, family, or social situation related to taking care of my illness. |  |  |  |  |  |
| 23. | Helped to make plans for how to get support from my friends, family or community. |  |  |  |  |  |
| 24. | Told how important the things I do to take care of my illness (e.g. exercise) were for my health. |  |  |  |  |  |
| 25. | Set a goal together with my team for what I could do to manage my condition. |  |  |  |  |  |
| 26. | Given a book or monitoring log in which to record the progress I am making. |  |  |  |  |  |

1. **What therapy goals has the attending physician worked out with you regarding therapy with opioid painkillers? (*Multiple answers are possible*.)**

|  | **No** therapy goals have been agreed | ***→If yes, please continue with question 25*** |
| --- | --- | --- |
|  | Optimal pain relief | |
|  | Avoiding worsening of the pain | |
|  | Enable participation in physiotherapy | |
|  | High safety with few side effects | |
|  | Avoidance of an addiction problem | |
|  | Participation in social life | |
|  | Employment | |
|  | “Being able to provide for yourself again” | |
|  | Improved sleep quality | |
|  | Other: ____________________ | |

1. Was a comprehensive treatment concept (e.g. physiotherapy, involvement of a psychotherapist) developed with you? If not, what do you think were the reasons that prevented the development of a comprehensive treatment concept?

|  | | Yes, there is a comprehensive treatment concept | |
| --- | --- | --- | --- |
|  | | No, there is no or no comprehensive treatment concept | |
|  | | **The following reasons stood in the way of developing a comprehensive treatment concept:**  **(*Multiple answers are possible*. )** | |
|  | | Lack of time of the doctor | |
|  | | Lack of expertise of the doctor | |
|  | | I have the feeling that the doctor does not respond to my needs | |
|  | | Difficulties in getting an appointment with a doctor | |
|  | | Doctor expresses financial constraints to prescribe further measures | |
|  | | I am being treated by several doctors and a coordination between the doctors  does not take place | |
|  | | Other: ____________________ | |

1. **The following is about the use of opioid** **painkillers in the last twelve months.**

- **Please make a cross *in each line.***

|  | **In the last 12 month** | | |
| --- | --- | --- | --- |
|  | **No** | **Yes, once** | **Yes, more than once** |
| Have you had any significant problems at work, school or in caring for your household related to taking opioid painkillers, such as absenteeism, poor performance, exclusion from school, neglect of children and household? |  |  |  |
| Were you under the influence of opioid painkillers when you were in situations with an increased risk of injury, e.g. in traffic or at work, when you were operating a machine or performing a dangerous activity? |  |  |  |
| Have you injured yourself unintentionally, i.e. had an accident or a serious fall, after taking opioid painkillers? |  |  |  |
| Have you had any legal problems related to opioid painkillers, e.g. possession of medication, theft or driving under the influence of medication? |  |  |  |
| Have your family or friends blamed you for taking opioid painkillers? |  |  |  |
| Did a relationship, e.g. with your partner, a family member or a friend, break up because of your use of opioid painkillers? |  |  |  |
| Have you got into financial difficulties because of your use of opioid painkillers? |  |  |  |
| Have you physically assaulted or injured someone while under the influence of opioid painkillers? |  |  |  |
| Have you taken opioid painkillers in larger quantities or for a longer period than prescribed or originally intended? |  |  |  |
| Have you experienced any discomfort when stopping opioid painkillers or reducing the dose? |  |  |  |
| Have you continued to take the same or a similar opioid painkiller to avoid discomfort when stopping or reducing the dose? |  |  |  |
| Did you find that you needed higher doses of opioid painkillers to achieve the desired effect or did taking the same amount for a longer period of time significantly weaken the effect of the medication? |  |  |  |
| Have you tried several times to reduce or stop taking opioid painkillers without success? |  |  |  |
| Did it take you a long time to get opioid painkillers (e.g. visited several doctors)? |  |  |  |
| Have you needed a lot of time to recover from the effects of opioid painkillers? |  |  |  |
| Have you restricted or given up important activities, e.g. your work or being with friends or relatives, because of taking opioid painkillers? |  |  |  |
| Did a relationship, e.g. with your partner, a family member or a friend, break up because of your use of opioid painkillers? |  |  |  |
| Have you got into financial difficulties because of your use of opioid painkillers? |  |  |  |
| Have you physically assaulted or injured someone while under the influence of opioid painkillers? |  |  |  |
| Have you taken opioid painkillers in larger quantities or for a longer period than prescribed or originally intended? |  |  |  |
| Have you experienced any discomfort or discomfort when stopping opioid painkillers or reducing the dose? |  |  |  |
| Have you continued to take the same or a similar opioid painkiller to avoid discomfort or discomfort when stopping or reducing the dose? |  |  |  |
| Did you find that you needed higher doses of opioid painkillers to achieve the desired effect or did taking the same amount for a longer period of time significantly weaken the effect of the medication? |  |  |  |
| Have you tried several times to reduce or stop taking opioid painkillers without success? |  |  |  |
| Did it take you a long time to get opioid painkillers (e.g. visited several doctors)? |  |  |  |
| Have you needed a lot of time to recover from the effects of opioid painkillers? |  |  |  |
| Have you restricted or given up important activities, e.g. your work or being with friends or relatives, because of taking opioid painkillers? |  |  |  |
| Have you taken opioid painkillers even though you knew that taking them would harm you? |  |  |  |
| Did a relationship, e.g. with your partner, a family member or a friend, break up because of your use of opioid painkillers? |  |  |  |
| Did you have such a strong craving for opioid painkillers that you could think of nothing else? |  |  |  |

1. **In which country were you and your parents born?**

| **I was born in** | | | |
| --- | --- | --- | --- |
|  | Germany |  | another country |
| **My mother was born in** | | | |
|  | Germany |  | another country |
| **My father was born in** | | | |
|  | Germany |  | another country |

1. **What is your highest educational or academic qualification?**

|  | Still in training or studying |  |
| --- | --- | --- |
|  | No training/ or study qualification |  |
|  | Vocational-in-company training / apprenticeship |  |
|  | Vocational school, commercial school |  |
|  | Technical school e.g. master craftsman, technician qualification |  |
|  | Degree (e.g. Bachelor, Master, Diploma, Doctorate) |  |

1. The term **family doctor** refers to doctors working in primary care (general practitioner/internist/general practitioner). [↑](#footnote-ref-1)
