# Supplementary material for: Patient-reported quality of outpatient healthcare in patients with chronic back or arthrosis pain with long-term opioid therapy in Germany
Source: BMC Prim Care. 2025 Jun 21;26:200. doi: 10.1186/s12875-025-02881-3 (PMC12181890; doi:10.1186/s12875-025-02881-3)
Supplement: Supplementary file 2 — Supplementary Material 2: Description of the variables used in the analysis. [file 12875_2025_2881_MOESM2_ESM.docx]

Additional file 2: Description of the variables used in the analysis.

| **variable** | **value specification** | **data source** | **reference to the question in the survey** |
| --- | --- | --- | --- |
| **Assessment of the quality of healthcare according to the chronic care model** | | | |
| PACIC-5A | 6 metric scales,  Minimum 1 (almost never)  Maximum 5 (almost always) | questionnaire, question 21 | German version of the Patient Assessment of Chronic Illness Care (PACIC) questionnaire (18) |
| 1. **patient characteristics** (see Error: Reference source not found) | | | |
| age | a. metric  b. ordinal  - 18-49 years  - 50-69 years  - 70-89 years  - ≥ 90 years | administrative claims data | not applicable |
| sex | -female  -male | administrative claims data | not applicable |
| migration background | -yes (at least one parent or insured person was not born in Germany)  -no | questionnaire, question 34 | self-developed |
| pain diagnosis | -back pain  -arthrosis pain  -both types of pain | questionnaire, question 1 | self-developed based on the guideline recommendations (II.1, II.2) (6) |
| highest educational qualification | -no degree  -currently at school/vocational training  -vocational training/apprenticeship  -technical school  -university degree | questionnaire, question 38 | self-developed |
| 1. **patient’s health situation** (see Error: Reference source not found) | | | |
| psychological distress - severity | -none (0-2 points)  -mild (3-5 points)  -moderate (6-8 points)  -severe (9-12 points) | questionnaire, question 18 | PHQ-4 (17, 19) |
| symptoms of opioid Substance Use Disorder | -none (0-1 criteria)  -mild (2-3 criteria)  -moderate (4-5 criteria)  -severe (6-9 criteria) | questionnaire, question 32; evaluation method see Error: Reference source not found | Excerpt from the German Epidemiological Addiction Survey 2015, answer options of question 27 of the original questionnaire were slightly adjusted (16) |
| intensity of pain – related impairments | -Grade 0 no pain  -Grade I low pain and low pain-related- impairment  -Grade II severely pain and low pain-related impairment  -Grade III severely pain-related-impairment, moderate limiting  -Grade IV severely pain-related-impairment, moderate limiting | questionnaire, questions 8, 9; response format of question 9 a) was modified | Graded Chronic Pain Scale Items and Scoring (25)  German questionnaire version (15)  Evaluation method (26) |
| 1. **pain treatment aspects** (see Error: Reference source not found) | | | |
| outpatient pain therapy | -yes  -no | questionnaire, question 10 | self-developed |
| setting of therapy goals | -yes  -no | questionnaire, question 22 | self-developed based on the guideline recommendations (III.8) (6) |
| comprehensive treatment concept | -yes  -no (none/non-comprehensive) | questionnaire, question 25 | self-developed based on the guideline recommendations (III.4) (6) |
| categorized procedures of interdisciplinary pain therapy | | | |
| 1. special medicinal pain management procedures | -yes  -no | questionnaire, question 13^[[1]](#footnote-2)^ | self-developed based on the guideline recommendations (III.4) (6) |
| 2. remedies | -yes  -no | questionnaire, question 13^[[2]](#footnote-3)^ |  |
| 3. psychotherapy | -yes  -no | questionnaire, question 13 |  |
| 4. day patient/inpatient procedures | -yes  -no | questionnaire, question 13^[[3]](#footnote-4)^ |  |
| 5. other non-medicinal complementary procedures | -yes  -no | questionnaire, question 13^[[4]](#footnote-5)^ |  |
| number of categories of procedures of interdisciplinary therapy | metric variable (number of the 5 procedure groups listed above)  minimum 0  maximum 5 | questionnaire, question 13 |  |

1. infusions; injection into the pain area, nerve blocks; injection at the spinal cord (e.g., epidural); spinal cord probe or pump systems [↑](#footnote-ref-2)
2. physiotherapy; manual therapy; massages, baths, cold/heat therapy [↑](#footnote-ref-3)
3. reconvalescence/rehabilitation treatment; outpatient/day patient/inpatient pain therapy; day clinic [↑](#footnote-ref-4)
4. transcutaneous electrical nerve stimulation (TENS), acupuncture, chiropractic, chiropractic (osteopathy) [↑](#footnote-ref-5)
