# Supplementary material for: Patient-reported quality of outpatient healthcare in patients with chronic back or arthrosis pain with long-term opioid therapy in Germany
Source: BMC Prim Care. 2025 Jun 21;26:200. doi: 10.1186/s12875-025-02881-3 (PMC12181890; doi:10.1186/s12875-025-02881-3)
Supplement: Supplementary file 3 — Supplementary Material 3: Assignment systematics of the questions of the epidemiological addiction survey 2015 to the DSM-V criteria and description of the evaluation systematics. [file 12875_2025_2881_MOESM3_ESM.docx]

Additional file 3: Assignment systematics of the questions of the epidemiological addiction survey 2015 to the DSM-V criteria and description of the evaluation systematics.

| **DSM-V Diagnostic Criterion (24)** | **DSM-V Diagnostic Criterion – Description (24)** | **Question – epidemiological addiction survey 2015 (related to the past 6 month) (16)** | **DSM-V Criterion is met if the question is answered with the following variable expression** |
| --- | --- | --- | --- |
| **5** | Substance use impairs ability to fulfill major obligations at work, school, or home. | Have you had significant problems at work, school, or taking care of your household related to taking opioid pain medications, such as absenteeism, low performance, exclusion from school, neglect of children and household? | -Yes, more than one time |
| **8** | **Recurrent** substance use in physically unsafe environments. | Were you under the influence of opioid pain medication when you were in situations with increased risk of injury, such as on the road or at work, operating a machine, or performing a dangerous activity? | -Yes, more than one time |
| **9** | **Persistent** substance use despite knowledge that it may cause or exacerbate physical or psychological problems. | Have you unintentionally injured yourself, i.e., had an accident or a serious fall, after taking opioid pain medications? | -Yes, more than one time |
| **6** | **Continued** use of the substance despite it causing significant social or interpersonal problems. | Have you had any legal problems related to opioid pain medications, such as possession of medications, theft, or driving under the influence of medications? | -Yes, one time  -Yes, more than one time |
| **6** | **Continued** use of the substance despite it causing significant social or interpersonal problems. | Have your family or friends blamed you for taking opioid pain medications? | -Yes, one time  -Yes, more than one time |
| **6** | **Continued** use of the substance despite it causing significant social or interpersonal problems. | Did a relationship, such as with your partner, a family member, or a friend, break up because of your use of opioid pain medications? | -Yes, one time  -Yes, more than one time |
| **6** | **Continued** use of the substance despite it causing significant social or interpersonal problems. | Do you have financial difficulties because of your use of opioid pain medications? | -Yes, one time  -Yes, more than one time |
| **6** | **Continued** use of the substance despite it causing significant social or interpersonal problems. | Have you physically assaulted or injured someone while influenced by opioid pain medications? | -Yes, one time  -Yes, more than one time |
| **1** | Consuming the substance in larger amounts and for a longer amount of time than intended. | Have you taken opioid pain medications in larger amounts or for a longer period of time than prescribed or originally intended? | -Yes, one time  -Yes, more than one time |
| **2** | **Persistent desire** to cut down or regulate use. The individual may have unsuccessfully attempted to stop in the past. | Have you tried several times to reduce or stop taking opioid pain medications to no avail? | -Yes, one time  -Yes, more than one time |
| **3** | Spending a great deal of time obtaining, using, or recovering from the effects of substance use. | Did it take a long time for you to get opioid pain medications (e.g., see several doctors)? | -Yes, one time  -Yes, more than one time |
| **3** | Spending a great deal of time obtaining, using, or recovering from the effects of substance use. | Have you needed a lot of time to recover from the effects of opioid pain medications? | -Yes, one time  -Yes, more than one time |
| **7** | Reduction or discontinuation of recreational, social, or occupational activities because of substance use. | Have you limited or given up important activities, such as your work or socializing with friends or family, because of taking opioid pain medications? | -Yes, one time  -Yes, more than one time |
| **9** | **Persistent** substance use despite knowledge that it may cause or exacerbate physical or psychological problems. | Have you taken opioid pain medications even knowing that taking them would harm you? | -Yes, more than one time |
| **4** | Experiencing craving, a pressing desire to use the substance. | Did you have such a strong desire or need for opioid pain medications that you could not resist it? | -Yes, one time  -Yes, more than one time |
| **4** | Experiencing craving, a pressing desire to use the substance. | Did you have such a strong desire for opioid pain medications that you could think of nothing else? | -Yes, one time  -Yes, more than one time |

Calculation: Criteria according to DSM-V are counted as fulfilled if at least one associated answer is given. For the classification of the severity of an opioid addiction problem, the number of fulfilled criteria is summed up.

Missing values: a DSM-V criterion is evaluated if at least one question related to this criterion is not missing. For the calculation of the sum score, the underlying nine criteria must not be missing.
