# Supplementary material for: Patient-reported quality of outpatient healthcare in patients with chronic back or arthrosis pain with long-term opioid therapy in Germany
Source: BMC Prim Care. 2025 Jun 21;26:200. doi: 10.1186/s12875-025-02881-3 (PMC12181890; doi:10.1186/s12875-025-02881-3)
Supplement: Supplementary file 4 — Supplementary Material 4: Descriptive results of the independent variables used in subgroup analysis. [file 12875_2025_2881_MOESM4_ESM.docx]

Additional file 4: Descriptive results of the independent variables used in subgroup analysis.

| **variable** | **total (n=661)** | | |  |
| --- | --- | --- | --- | --- |
|  | **n** | **valid %** | |  |
| 1. **patient characteristics** | | | |  |
| age group |  | |  |  |
| 18-49 years | 41 | 6.2 | |  |
| 50-69 years | 274 | 41.5 | |  |
| 70-89 years | 327 | 49.4 | |  |
| ≥ 90 years | 19 | 2.9 | |  |
| sex |  |  | |  |
| female | 499 | 75.5 | |  |
| male | 162 | 24.5 | |  |
| migration background |  |  | |  |
| no | 578 | 88.4 | |  |
| yes | 76 | 11.6 | |  |
| sum | 654 | 100 | |  |
| pain diagnosis |  |  | |  |
| back pain | 209 | 31.6 | |  |
| arthrosis pain | 95 | 14.4 | |  |
| both types of pain | 357 | 54.0 | |  |
| highest educational qualification |  |  | |  |
| no degree | 27 | 4.4 | |  |
| vocational training/apprenticeship | 448 | 72.3 | |  |
| technical school | 74 | 12.0 | |  |
| university degree | 70 | 11.3 | |  |
| sum | 619 | 100 | |  |
| 1. **patient’s health situation** | | | |  |
| psychological distress - severity |  |  | |  |
| none | 199 | 35.4 | |  |
| low | 169 | 30.1 | |  |
| moderate | 123 | 21.9 | |  |
| severe | 71 | 12.6 | |  |
| sum | 562 | 100 | |  |
| symptoms of opioid Substance Use Disorder |  |  | |  |
| none | 331 | 56.8 | |  |
| mild | 143 | 24.6 | |  |
| moderate | 64 | 11.0 | |  |
| severe | 44 | 7.5 | |  |
| sum | 582 | 100 | |  |
| intensity of pain-related impairment – graded chronic pain scale |  |  | |  |
| no pain | 16 | 3.0 | |  |
| low pain and low pain-related- impairment | 24 | 4.4 | |  |
| severely pain and low pain-related impairment | 41 | 7.6 | |  |
| severely pain-related-impairment, moderate limiting | 165 | 30.4 | |  |
| severely pain-related-impairment, severely limiting | 296 | 54.6 | |  |
| sum | 542 | 100 | |  |
| 1. **pain treatment aspects** | | | |  |
| outpatient pain therapy |  |  | |  |
| yes | 369 | 56.4 | |  |
| no | 285 | 43.6 | |  |
| sum | 654 | 100 | |  |
| setting of therapy goals |  |  | | |
| yes | 437 | 71.2 | | |
| no | 177 | 28.8 | | |
| sum | 614 | 100 | | |
| comprehensive treatment concept |  |  | | |
| yes | 186 | 32.3 | | |
| no (none/not comprehensive) | 390 | 67.7 | | |
| sum | 576 | 100 | | |
| use of interdisciplinary pain therapy^a^ (n=655) |  |  | | |
| special medicinal pain management procedures | 447 | 68.2 | | |
| remedies | 579 | 88.4 | | |
| psychotherapy | 216 | 33.0 | | |
| day patient/inpatient procedures | 443 | 67.6 | | |
| other non-medicinal complementary procedures | 393 | 60.0 | | |
| sum | 2,078 | 317.3 | | |
| number of categories of procedures of interdisciplinary therapy |  |  | | |
| 0 | 36 | 5.5 | | |
| 1 | 72 | 11.0 | | |
| 2 | 97 | 14.8 | | |
| 3 | 126 | 19.2 | | |
| 4 | 186 | 28.4 | | |
| 5 | 138 | 21.1 | | |
| sum | 655 | 100 | | |

^a^ multiple response was possible.
