# Supplementary material for: Patient-reported quality of outpatient healthcare in patients with chronic back or arthrosis pain with long-term opioid therapy in Germany
Source: BMC Prim Care. 2025 Jun 21;26:200. doi: 10.1186/s12875-025-02881-3 (PMC12181890; doi:10.1186/s12875-025-02881-3)
Supplement: Supplementary file 5 — Supplementary Material 5: Subgroup sizes of mean value calculation of the Patient Assessment of Chronic Illness Care (PACIC-5A) subscores. [file 12875_2025_2881_MOESM5_ESM.docx]

Additional file 5: Subgroup sizes of mean value calculation of the Patient Assessment of Chronic Illness Care (PACIC-5A) subscores.

| **independent variables** | **value** | **assess** | **advise** | **agree** | **assist** | **arrange** | **5A summary score** |
| --- | --- | --- | --- | --- | --- | --- | --- |
|  |  | **n** | **n** | **n** | **n** | **n** | **n** |
| **age** | 18-49 | 41 | 40 | 41 | 41 | 41 | 40 |
|  | 50-69 | 254 | 252 | 255 | 250 | 252 | 246 |
|  | 70-89 | 274 | 266 | 273 | 276 | 270 | 252 |
|  | ≥ 90 | 15 | 15 | 15 | 15 | 15 | 14 |
|  | **total** | **584** | **573** | **584** | **582** | **578** | **552** |
| **highest educational qualification** | currently at school/vocational training | 0 | 0 | 0 | 0 | 0 | 0 |
|  | no degree | 23 | 21 | 21 | 22 | 22 | 19 |
|  | vocational training/apprenticeship | 402 | 394 | 402 | 399 | 398 | 379 |
|  | technical school | 64 | 65 | 67 | 66 | 64 | 63 |
|  | university degree | 66 | 67 | 66 | 67 | 66 | 65 |
|  | **total** | **555** | **547** | **556** | **554** | **550** | **526** |
| **psychological distress - severity** | none | 185 | 180 | 181 | 181 | 181 | 176 |
|  | low | 155 | 155 | 156 | 155 | 155 | 150 |
|  | moderate | 118 | 119 | 121 | 119 | 117 | 116 |
|  | severe | 68 | 66 | 67 | 69 | 68 | 64 |
|  | **total** | **526** | **520** | **525** | **524** | **521** | **506** |
| **symptoms of opioid Substance Use Disorder** | none | 299 | 296 | 297 | 300 | 294 | 284 |
|  | low | 131 | 130 | 133 | 131 | 132 | 127 |
|  | moderate | 61 | 60 | 61 | 61 | 59 | 59 |
|  | severe | 43 | 43 | 42 | 43 | 43 | 42 |
|  | **total** | **534** | **529** | **533** | **535** | **528** | **512** |
| **intensity of pain-related impairment** | no pain | 15 | 15 | 14 | 15 | 15 | 14 |
|  | low pain and low pain-related- impairment | 24 | 23 | 22 | 23 | 23 | 22 |
|  | severely pain and low pain-related impairment | 36 | 36 | 38 | 36 | 38 | 33 |
|  | severely pain-related-impairment, moderate limiting | 147 | 150 | 151 | 152 | 147 | 144 |
|  | severely pain-related-impairment, severely limiting | 279 | 271 | 274 | 277 | 276 | 265 |
|  | **total** | **501** | **495** | **499** | **503** | **499** | **478** |
| **outpatient pain therapy** | yes | 340 | 332 | 339 | 342 | 335 | 319 |
|  | no | 241 | 238 | 242 | 237 | 240 | 230 |
|  | **total** | **581** | **570** | **581** | **579** | **575** | **549** |
| **setting of therapy goals** | yes | 395 | 387 | 396 | 396 | 391 | 371 |
|  | no | 162 | 160 | 162 | 160 | 162 | 158 |
|  | **total** | **557** | **547** | **558** | **556** | **553** | **529** |
| **comprehensive treatment concept** | yes | 366 | 366 | 364 | 369 | 362 | 350 |
|  | no (none/not comprehensive) | 167 | 163 | 170 | 162 | 167 | 158 |
|  | **total** | **533** | **529** | **534** | **531** | **529** | **508** |
| **special medicinal pain management procedures** | yes | 408 | 402 | 409 | 409 | 403 | 391 |
|  | no | 173 | 169 | 172 | 171 | 172 | 159 |
|  | **total** | **581** | **571** | **581** | **580** | **575** | **550** |
| **remedies** | yes | 520 | 514 | 521 | 522 | 516 | 497 |
|  | no | 61 | 57 | 60 | 58 | 59 | 53 |
|  | **total** | **581** | **571** | **581** | **580** | **575** | **550** |
| **psychotherapy** | yes | 200 | 197 | 203 | 202 | 199 | 191 |
|  | no | 381 | 374 | 378 | 378 | 376 | 359 |
|  | **total** | **581** | **571** | **581** | **580** | **575** | **550** |
| **day patient/inpatient procedures** | yes | 406 | 400 | 406 | 406 | 403 | 386 |
|  | no | 175 | 171 | 175 | 174 | 172 | 164 |
|  | **total** | **581** | **571** | **581** | **580** | **575** | **550** |
| **other non-medicinal complementary procedures** | yes | 361 | 356 | 363 | 361 | 359 | 349 |
|  | no | 220 | 215 | 218 | 219 | 216 | 201 |
|  | **total** | **581** | **571** | **581** | **580** | **575** | **550** |
| **number of categories of procedures of interdisciplinary pain therapy** | 0 | 29 | 28 | 29 | 27 | 29 | 27 |
|  | 1 | 63 | 60 | 60 | 61 | 60 | 56 |
|  | 2 | 76 | 75 | 76 | 78 | 75 | 68 |
|  | 3 | 105 | 105 | 107 | 107 | 106 | 102 |
|  | 4 | 175 | 171 | 176 | 173 | 173 | 169 |
|  | 5 | 133 | 132 | 133 | 134 | 132 | 128 |
|  | **total** | **581** | **571** | **581** | **580** | **575** | **550** |
| **sex** | female | 436 | 424 | 443 | 433 | 430 | 406 |
|  | male | 148 | 149 | 151 | 149 | 148 | 146 |
|  | **total** | **584** | **573** | **584** | **582** | **578** | **552** |
| **migration background** | yes | 66 | 66 | 67 | 66 | 66 | 63 |
|  | no | 513 | 502 | 512 | 511 | 507 | 484 |
|  | **total** | **579** | **568** | **579** | **577** | **573** | **547** |
| **pain diagnosis** | back pain | 181 | 177 | 181 | 179 | 177 | 169 |
|  | arthrosis pain | 80 | 81 | 82 | 81 | 82 | 77 |
|  | both types of pain | 323 | 315 | 321 | 322 | 319 | 306 |
|  | **total** | **584** | **573** | **584** | **582** | **578** | **552** |
