# Supplementary material for: Patient-reported quality of outpatient healthcare in patients with chronic back or arthrosis pain with long-term opioid therapy in Germany
Source: BMC Prim Care. 2025 Jun 21;26:200. doi: 10.1186/s12875-025-02881-3 (PMC12181890; doi:10.1186/s12875-025-02881-3)
Supplement: Supplementary file 6 — Supplementary Material 6: Subgroup analysis of the scores of the 5A model of the Patient Assessment of Chronic Illness Care (PACIC-5A) on migration background, sex and pain diagnosis. [file 12875_2025_2881_MOESM6_ESM.docx]

Additional file 6: Subgroup analysis of the scores of the 5A model of the Patient Assessment of Chronic Illness Care (PACIC-5A) on migration background, sex and pain diagnosis.

| **independent variables** | **value** | **assess** | | **advise** | | **agree** | | **assist** | | **arrange** | | **5A summary score** | |
| --- | --- | --- | --- | --- | --- | --- | --- | --- | --- | --- | --- | --- | --- |
|  |  | X̅ | Eta | X̅ | Eta | X̅ | Eta | X̅ | Eta | X̅ | Eta | X̅ | Eta |
| **migration background** | **yes** | 2.6955 | 0.009 | 2.4917 | 0.032 | 2.7582 | 0.004 | 1.9152 | 0.002 | 1.9152 | 0.007 | 2.4077 | 0.013 |
|  | **no** | 2.6644 |  | 2.4069 |  | 2.7449 |  | 1.9103 |  | 1.8959 |  | 2.3728 |  |
| **sex** | **male** | 2.7905 | 0.060 | 2.5802 | 0.113 | 2.9017 | 0.079 | 1.9951 | 0.055 | 1.9595 | 0.040 | 2.4747 | 0.066 |
|  | **female** | 2.6334 |  | 2.3596 |  | 2.6982 |  | 1.8857 |  | 1.8809 |  | 2.3456 |  |
| **pain diagnosis** | **back** | 2.6641 | 0.005 | 2.3746 | 0.051 | 2.7912 | 0.041 | 1.9269 | 0.055 | 1.9232 | 0.046 | 2.3806 | 0.039 |
|  | **arthrosis** | 2.6800 |  | 2.3568 |  | 2.6427 |  | 1.7951 |  | 1.8067 |  | 2.2998 |  |
|  | **both** | 2.6766 |  | 2.4562 |  | 2.7556 |  | 1.9362 |  | 1.9130 |  | 2.3994 |  |

* p<0.05 (test for significance of group differences: Mann-Whitney U test (migration, gender), Kruskal-Wallis test (pain diagnosis), Spearman-Rho (age (metric))

X̅ mean of the PACIC-5A scores
